# Chordoma cancer stem cell subpopulation characterization may guide targeted immunotherapy approaches to reduce disease recurrence

**DOI:** 10.3389/fonc.2024.1376622

**Published:** 2024-04-29

**Authors:** Diana C. Lopez, Kellsye P. Fabian, Michelle R. Padget, Yvette L. Robbins, Joshua T. Kowalczyk, Wiem Lassoued, Danielle M. Pastor, Clint T. Allen, Gary L. Gallia, James L. Gulley, James W. Hodge, Nyall R. London

**Affiliations:** ^1^ Sinonasal and Skull Base Tumor Program, Surgical Oncology Program, Center for Cancer Research, National Cancer Institute, National Institutes of Health, Bethesda, MD, United States; ^2^ Department of Otolaryngology-Head and Neck Surgery, Johns Hopkins University School of Medicine, Baltimore, MD, United States; ^3^ Center for Immuno-Oncology, National Cancer Institute, National Institutes of Health, Bethesda, MD, United States; ^4^ Center for Cancer Research, National Cancer Institute, National Institutes of Health, Bethesda, MD, United States; ^5^ Head and Neck Section, Surgical Oncology Program, Center for Cancer Research, National Cancer Institute, National Institutes of Health, Bethesda, MD, United States; ^6^ Department of Medicine, Duke University School of Medicine, Durham, NC, United States; ^7^ Department of Neurosurgery, Johns Hopkins University School of Medicine;, Baltimore, MD, United States

**Keywords:** chordoma, cancer stem cells (CSC), tumor microenvironment (TME), immunotherapy, PD-L1

## Abstract

**Introduction:**

Cancer stem cells (CSCs), a group of tumor-initiating and tumor-maintaining cells, may be major players in the treatment resistance and recurrence distinctive of chordoma. Characterizing CSCs is crucial to better targeting this subpopulation.

**Methods:**

Using flow cytometry, six chordoma cell lines were evaluated for CSC composition. In vitro, cell lines were stained for B7H6, HER2, MICA-B, ULBP1, EGFR, and PD-L1 surface markers. Eighteen resected chordomas were stained using a multispectral immunofluorescence (mIF) antibody panel to identify CSCs in vivo. HALO software was used for quantitative CSC density and spatial analysis.

**Results:**

In vitro, chordoma CSCs express more B7H6, MICA-B, and ULBP1, assessed by percent positivity and mean fluorescence intensity (MFI), as compared to non-CSCs in all cell lines. PD- L1 percent positivity is increased by >20% in CSCs compared to non-CSCs in all cell lines except CH22. In vivo, CSCs comprise 1.39% of chordoma cells and most are PD-L1+ (75.18%). A spatial analysis suggests that chordoma CSCs cluster at an average distance of 71.51 mm (SD 73.40 mm) from stroma.

**Discussion:**

To our knowledge, this study is the first to identify individual chordoma CSCs and describe their surface phenotypes using in vitro and in vivo methods. PD-L1 is overexpressed on CSCs in chordoma human cell lines and operative tumor samples. Similarly, potential immunotherapeutic targets on CSCs, including B7H6, MICA-B, ULBP1, EGFR, and HER2 are overexpressed across cell lines. Targeting these markers may have a preferential role in combating CSCs, an aggressive subpopulation likely consequential to chordoma’s high recurrence rate.

## Introduction

Chordomas are rare, heterogeneous, notochord-derived malignancies of bone. These originate within the skull base (30%), vertebral spine (20%), and sacrum/coccyx (50%) (1–5). Chordomas comprise 1-4% of bone malignancies and possess a low annual tumor incidence of approximately 1 per 1,000,000 individuals (3, 4, 6, 7). Although they display patterns of indolent growth, chordomas expand in a locally destructive manner (2, 6), for which treatment options remain limited. Standard of care currently consists of surgical resection when feasible and radiotherapy (2). However, radical resection, our most effective treatment (8), is difficult to achieve, particularly when extricating skull base tumors due to a risk of damaging important, nearby neurovascular structures including cranial nerves and the brainstem (1, 2). Residual tumor cells render disease relapse likely (1). Furthermore, chordomas possess well-described characteristics of chemoradiation resistance and a high recurrence propensity (2, 6). Recurrence is deemed the most important factor in patient mortality following diagnosis (6, 9). Metastasis results in 3-30% of cases, most often in the context of a recurrent tumor (2, 10, 11). Cancer stem cells have been implicated in resistance to common therapies, recurrence, and metastasis (12–14), and thus, further investigation into this subpopulation may generate new angles from which to mitigate chordoma treatment failure.

The cancer stem cell (CSC) population is a group of tumor-initiating and tumor-maintaining cells with properties of self-renewal, *de novo* cancer formation following orthotopic implantation, and multi-lineage differentiation (12–17). To be accurately classified as such, CSCs should exhibit functional tumorigenic behavior, including colony- and tumorosphere- formation, increased migration, and enhanced invasion. CD15^+^CD133^+^ chordoma cells were recently were shown to meet these criteria in a 2019 study by Tuysuz et al (18). Prior to this, chordoma cells with enhanced CD15^+^CD133^+^ expression had been already been shown to grow anchorage-independent colonies (19). Importantly, despite the low prevalence of this cancer subpopulation (17), CSCs are implicated in radioresistance, chemoresistance, and metastasis (20, 21). CSCs have been shown to promote tumor formation in a host of other cancers including glioblastoma, myeloid malignancies, melanoma, and cancers of the breast, lung, colon, and pancreas (15, 17, 21–25). However, the expression of surface markers that define CSCs are tissue-type specific (17). Prior chordoma studies have illustrated that CSCs may be defined by surface expression of various combinations of markers, including CD15, CD24, CD133, and ALDH (15, 19, 20, 25). Investigation into the targeting of CSCs has been ongoing for years, using a range of strategies from focusing on stem cell associated pathways, such as the Wnt/β-catenin and Notch pathways, to immunologic techniques like adoptive cell transfer and checkpoint blockade, and even chimeric antigen receptor (CAR) T cell therapy directed at CD133^+^ CSCs (13, 16). However, much progress remains to be seen in the study of chordoma CSCs.

Herein we build upon work previously published by our group that identified chordoma CSCs with flow cytometry in four cell lines and showed that CSCs could be eliminated by avelumab (PD-L1 inhibitor)-mediated antibody dependent cellular cytotoxicity (ADCC) (20). We also demonstrated significantly increased surface expression of NK-activating receptor ligand B7H6 and PD-L1 in chordoma CSCs as compared to non-CSCs within a single cell line, UM-Chor1 (15). Our latter study went as far as to show preferential CSC vulnerability to a combinatorial treatment approach of natural killer cells, an anti-PD-L1 agent, and an IL-15 superagonist intended to induce NK- and T-cell compartment killing effects (15). These investigations provide initial evidence for the ability to enhance elimination of tumor-initiating cells in chordoma. To prime the development of efficacious molecular targeted therapy for decreasing CSC burden in chordoma, a better characterization of CSC behavior and phenotypic expression, or of viable immunotherapeutic targets found on CSCs, must be achieved.

With this study, we characterize chordoma CSCs by phenotypic surface marker expression and spatial distribution in two contexts, *in vitro* using flow cytometry and *in vivo* using multispectral immunofluorescence. We hypothesized that all chordoma cell lines examined *in vitro* would preferentially express immunotherapeutic target surface ligands in a subpopulation of CSC marker-enriched cells. Multispectral immunofluorescence (mIF) is an imaging method by which we may individually identify cells of interest within the tumor microenvironment, detect protein coexpression on these cells, and understand the spatial relationship among these (26). We proposed that using mIF, we could detect the presence and relative location of CSCs within operative chordoma samples. Such efforts to better characterize and later develop targeted therapy against chordoma CSCs may pave a path worth pursuing for improved control against recurrent and lethal disease.

## Materials and methods

### Cell lines and culture

Six chordoma cell lines were cultured, harvested, stained, and analyzed via flow cytometry for CSC burden and characterization. These include JHC7, UMChor1, and CH22 from the Chordoma Foundation, and UCH1, MugChor1, and UCH17M from ATCC.

UMChor1, UCH1, UCH17M, and MugChor1 were cultured in 4:1 IMDM : RPMI, CH22 in RPMI, and JHC7 in DMEM:F12 complete growth media. All complete growth medias contained 10% fetal bovine serum, 1% penicillin-streptomycin, 1% nonessential amino acids, 1% L-glutamine, 1% hepes buffer, and 1% sodium pyruvate. Cells in culture had medium changed twice per week and were passaged after reaching 80% confluency. All cells harvested for staining were at a passage number 45 or below and at 92% viability or greater. All cell lines were serially verified to be negative for *Mycoplasma* infection with the Lonza Bioscence two-step luminescence assay.

### Flow cytometric analysis of surface markers

For each experiment, cells were trypsinized and harvested from a single flask to yield 200,000 cells in 100 µL of cell line-appropriate media. 400,000 cells per well were plated onto a 96-well plate. Cells were washed in phosphate buffered saline (PBS). Cells were labeled withLIVE/DEAD Fixable Aqua Dead Cell Stain Kit for 405 nm excitation (ThermoFisher, L34957) according to the manufacturer’s protocol. 96-well plates were incubated on ice and in the dark for 20 minutes. All wells were washed and resuspended in 200 µL of FACS buffer (PBS + 1% BSA).

Cells were stained for nine antibodies of interest including CD15, CD24, CD133, B7H6, HER2, MICA-B, ULBP1, EGFR, and PD-L1. Marker staining was divided into two panels. Panel A consisted of anti-human CD15-PE (BD Biosciences, 562371), CD24-BV711 (BD Biosciences, 563401), CD133-APC (BD Biosciences, 566596), B7H6-AF700 (R&D Systems, FAB7144N-100UG), HER2-BV786 (BD Biosciences, 744747), and MICA/B-PECy7 (BioLegend, 320917). Panel B consisted of anti-human CD15-PE (BD Biosciences, 562371), CD24-BV711 (BD Biosciences, 563401), CD133-APC (BD Biosciences, 566596), ULBP1-AF700 (R&D Systems, FAB1380N-100UG), EGFR-BV786 (BD Biosciences, 742606), PD-L1-PECy7 (BioLegend, 124313). Isotypes used were PE Mouse IgG1 k Isotype Control (BioLegend, 981804), Mouse IgG2a, k BV711 Isotype Control (BD Biosciences, 563345), APC Mouse IgG1 k Isotype Control (BioLegend, 400119) Mouse IgG1 Alexa Fluor 700-conjugate isotypes (R&D Systems, IC002N), Mouse IgG1, k BV786 Isotype Control (BD Biosciences, 563330), Mouse IgG2a, k PECy7 Isotype Control (BioLegend, 400232), Mouse IgG2a Alexa Fluor 700-conjugate isotypes (R&D Systems, IC003N), Mouse IgG2b, k BV786 Isotype Control (BD Biosciences, 564090), Rat IgG2b, k PECy7 Isotype Control (BioLegend, 400617).

Human TruStain FcX Fc Receptor Blocking Solution (BioLegend, 422302) diluted 1:100 in FACS was applied to all wells except unstained and incubated for 10 minutes on ice. 5 µL of each relevant antibody or isotype listed above was added with sufficient FACS solution to total a 100 µL volume per well using a master mix. Following a 30-minute dark incubation on ice and two washes with FACS, 200 µL of Cytofix solution was added to all wells. Cells in Cytofix underwent a dark 30-minute incubation on ice and washed twice with FACS. All wells were filled with 200 µL FACS, and plates were wrapped in aluminum foil for storage until flow cytometry was run, no later than one week following staining. Flow cytometry was performed on BD LSRFortessa (BD Bioscences).

All experiments were completed in technical triplicate. Each experiment was conducted on three independent occasions to ensure consistent results.

Flow cytometry data was analyzed using FlowJo software v10.8.1. Single cells were gated to exclude clumping, and dead cells were excluded. Cells double positive for CD24 and CD133 were defined as CSCs while all other cells were called non-CSCs (**Supplementary Figure 1**). All samples within an experiment were down-sampled to the minimum number of CSCs identified to ensure standardization between groups. Positivity thresholds for each antibody marker were based off isotypes. Isotype controls consisted of vials with both CSCs and non-CSCs, a population that is representative of a typical chordoma tumor makeup. Percent positivity and mean fluorescence intensity (MFI) were compared for all antibodies in CSC and non-CSC groups. Percent positivity and MFI of technical triplicates were averaged for each experiment. Values for CSCs and non-CSCs were compared using a student’s t-test with a significance threshold of *p <*0.05. All statistics and were conducted and graphs prepared using GraphPad Prism version 9.2.

### Multispectral immunofluorescence

Multispectral immunofluorescence technique was used for staining and quantification of *in vivo* CSCs within 18 resected chordoma tumors per the protocol described by Lopez et al. (26), summarized below.

### Patient population

Formalin fixed paraffin embedded (FFPE) chordoma sections were obtained from 20 patients, 10 from the Chordoma Foundation Biobank and 10 from Johns Hopkins University. Clinical variables collected were patient demographics (age, sex, race, and ethnicity), anatomic site of tumor origin (skull base, spine, sacrum/coccyx), disease stage, and whether radiotherapy treatment regimen was administered.

### Panel validation

Each antibody’s optimal dilution for chordoma tissue staining was first determined with monoplex immunohistochemistry (IHC) and then monoplex immunofluorescence (IF) as described (26). Normal tonsil or normal lung tissue (4-5µm sections) served as positive controls and unstained chordoma tissue (4-5µm sections) served as a negative control for validation and staining steps. Multiple antibody combinations were evaluated to determine the most appropriate staining order while building a validated CSC multispectral immunofluorescence (MIF) panel.

The CSC panel was comprised of antibodies against CD24 (Novus Biologicals [ML5], #NB100-77903; 1:1000), ALDH1 (Abcam [EP1933Y], #ab52492; 1:400), PD-L1 (Cell Signaling [E1L3N(R)], #13684S; 1:300), CD15 (BD Biosciences [HI98], #555400; 1:1500), and Cytokeratin (Santa Cruz [AE1/AE3], #sc-81714; 1:400) (**Supplementary Table 1**). This panel allowed for the recognition of CSCs *in vivo*, defined as CD15^+^, CD24^+^, ALDH1^+^, cytokeratin^+^ chordoma cells. CD133 was excluded from this analysis due to unreliable, nonspecific staining of the antibody despite the attempted validation of several versions of the antibody at a variety of dilutions. All slides were counterstained with 4′,6-diamidino-2-phenylindole (DAPI) to identify cell nuclei. Cytokeratin distinguished chordoma tumor cells.

### Tissue preparation, staining, and scanning

Slides were prepared, stained, and scanned per the previously described protocol (26). Leica BOND Rx autostainer (Leica Biosystems Melbourne Pty Ltd, Melbourne, Australia) was used for the deparaffinization and staining of all tissue. Heat induced epitope retrieval (HIER) was completed for all antibodies except CD15. Primary antibody, secondary HRP-conjugated antibody, and fluorescent signal amplification were applied to slides. OPAL (Akoya Biosciences) multiplex kit consisting of OPAL-520, 570, 620, 690, 780 conjugates were used to study five simultaneous antibodies. Slides were cover slipped with the Leica CV5030 automated glass cover slipper (Leica Biosystems, Nussloch, Germany Ltd) and high-resolution digital images were produced at 40x magnification using PerkinElmer Vectra Polaris.

### Image analysis

Images were analyzed with the HALO^®^ (Indica Labs, Albuquerque, NM, USA) platform v3.3. Slides were annotated into tumor parenchyma and stroma using the HALO^®^ random forest classifier based on cytokeratin staining. Areas manually excluded from the analysis were those containing bone, bone marrow, blood vessels, and auto fluorescent tissue subsections. Individual biomarker fluorescence intensity thresholds were set to denote cells of each phenotype of interest. These were calibrated for each independent specimen to account for staining uptake variability. The HALO^®^ Highplex FL analysis algorithm v4.1.3 was used for CSC quantification analysis. Spatial relationships between CSCs and stromal edges were evaluated using infiltration analysis. HALO^®^ density heat maps of all CSC subpopulations were created to visually compare morphologic patterns within the tumors.

### Statistical analysis

*In vivo* CSC PD-L1 percent positivity was represented with bar graphs. Mann Whitney tests were used to detect statistical differences in CSC cell density and PD-L1 positivity by age and exposure to radiotherapy. Kruskal Wallis tests were used to detect statistical differences in CSC cell density and PD-L1 positivity by anatomic site of origin and disease stage. A *p* value significance threshold of <0.05 was employed in all cases. All statistics were conducted and graphs prepared using GraphPad Prism version 9.2.

### Study approval

Clinical data was acquired from the Chordoma Foundation following National Institutes of Health Institutional Review Board exemption and via a retrospective chart review of Johns Hopkins University chordoma patients following approval by the Johns Hopkins Institutional Review Board.

## Results

Cancer stem cells (CSCs) have been associated with tumor recurrence and resistance to chemoradiation (20, 21) in a number of malignancies. Prior chordoma studies have demonstrated that CD15, CD24, CD133, and ALDH are markers for tumorigenic CSCs (18–20, 25). First, chordoma cell lines were investigated *in vitro* using flow cytometry for CSC density and characteristics. CSCs were defined using dual positive criteria for CD24 and CD133 expression based on a prior *in vitro* designation of the residential CSC population using this signature (20). Except for CH22, all chordoma cell lines had less than 5% CSC burden (SD 0.002-0.016). CH22, a recurrent, metastatic chordoma cell line of sacral origin, had the highest proportion of CSCs, with CSCs representing 16.98% (SD 0.014) of the cell population (**Table 1**).

**Table 1:** CSC burden by chordoma cell line.

Cell Line	Disease Status	%CSC	
		Mean	SD
CH22	Recurrent, metastatic	16.98%	0.014
JHC7	Primary	1.73%	0.004
MugChor1	Recurrent	2.32%	0.002
UCH1	Recurrent	4.08%	0.016
UCH17M	Metastatic	1.45%	0.003
UMChor1	Primary	1.38%	0.002

Six chordoma cell lines, CH22, JHC7, MugChor1, UCH1, UCH17M, and UMChor1 were assessed using flow cytometry for CSC burden (mean, SD). CSCs were defined as CD24^+^CD133^+^ chordoma cells. CSCs, cancer stem cells.

**Figure 1:**
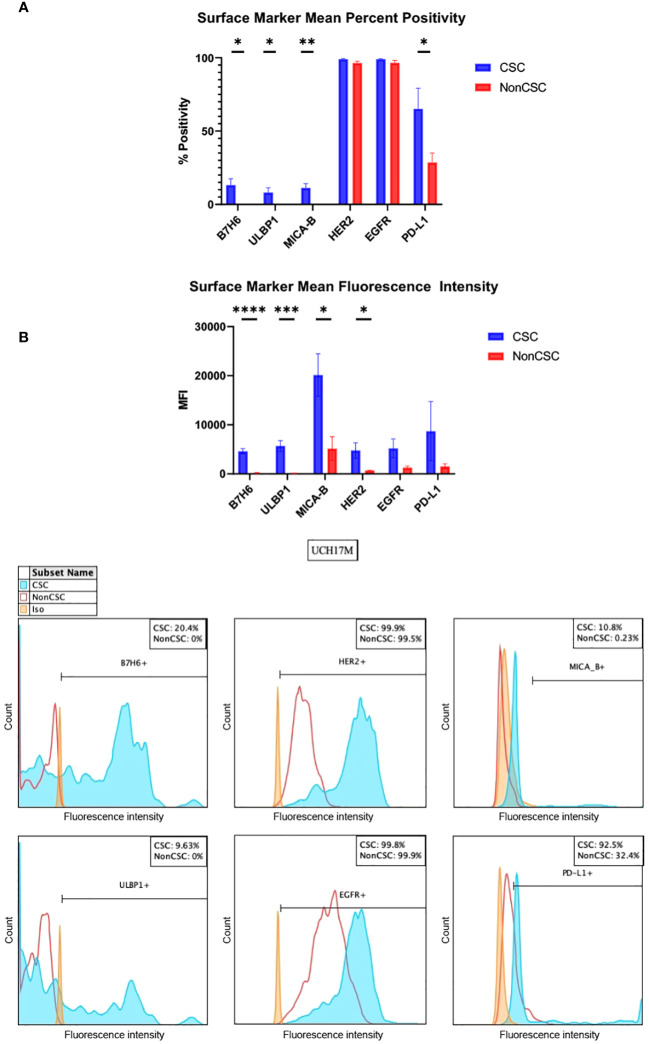
*In vitro* chordoma CSC characterization by surface markers of B7H6, ULBP1, MICA-B, HER2, EGFR, and PD-L1. **(A)** Surface markers are measured both by percent positivity (top) and MFI (bottom). Average values across six cell lines, collected via flow cytometry, are shown. Values for CSCs (blue) and NonCSCs (red) were compared using student’s t tests with a significance threshold of p <0.05. **(B)** Representative histograms of one technical triplicate in a UCH17M chordoma cell line experiment characterizing B7H6, ULBP1, HER2, EGFR, MICA-B, and PD-L1 surface marker percent expression in CSCs versus NonCSCs. Isotypes for each antibody were used to set positivity threshold. All chordoma cell line (n=6) flow cytometry experiments were analyzed using FlowJo software v10.8.1 in this manner. Each experiment was conducted in technical triplicate, and results were representative of three independent experiments. *p ≤ 0.05, **p ≤ 0.01, ***p ≤ 0.001, ****p ≤ 0.0001. CSC, cancer stem cells; MFI, mean fluorescence intensity.

B7H6, UL16 binding protein 1 (ULBP1), and MHC class I chain-related protein A and B (MICA-B) are ligands for activating receptors on natural killer cells (27–30). B7H6 was overexpressed in CSCs as compared to non-CSCs in all cell lines, evidenced both by measures of percent positivity and mean fluorescence intensity (MFI) (**Figure 1**, **Supplementary Table 2**). B7H6 expression was increased by >20% in the CSC group in cell lines CH22 and UCH17M (**Supplementary Table 3**). The mean B7H6 percent positivity of chordoma cells across cell lines was 13.2% in the CSC group and 0% in the non-CSC group (*p <*0.05) (**Figure 1**, **Supplementary Table 2**). The B7H6 antibody MFI detected across chordoma cell lines was 4,606 in the CSC group and 180 in the nonCSC group (*p <*0.0001) (**Supplementary Table 2**). Similarly, ULBP1 was overexpressed in CSCs at a mean of 8.2% compared to 0% in non-CSCs (*p <*0.05) and at a MFI of 5691 in CSCs compared to 111 in non-CSCs (*p <*0.001) (**Figure 1**, **Supplementary Table 2**). This trend also held true for the MICA-B antigen, which had increased expression in CSCs, mean of 11.3% in CSCs and 0.1% in non-CSCs (*p <*0.01) and MFI of 20,146 in CSCs and 5,167 in non-CSCs (*p <*0.05) (**Figure 1**, **Supplementary Table 2**).

Human epidermal growth factor receptor 2 (HER2) and epidermal growth factor receptor (EGFR) are tyrosine kinases of the epidermal growth factor receptor family, and mutations of these are implicated in the development of multiple cancers (31). Both tyrosine kinases were uniformly highly expressed as measured by percent positivity in CSCs (HER2 mean 99.0%, EGFR mean 99.0%) and non-CSCs (HER2 mean 96.5%, EGFR mean 96.5%). However, the MFI of these tyrosine kinases was increased in the CSC group (HER2 mean 4,775, EGFR mean 5,209) as compared to the non-CSC group (HER2 mean 708, EGFR mean 1,277). The only statistically significant comparison in this series was HER2 MFI (*p <*0.05) (**Figure 1**, **Supplementary Table 2**).

Programmed cell death ligand 1 (PD-L1) forms part of the PD-1/PD-L1 pathway exploited by neoplasms to evade immune surveillance, and its inhibition has been shown to be clinically efficacious against a number of cancers (26, 32, 33). CSCs, as compared to non-CSCs, overexpressed PD-L1 in all cell lines except CH22 (**Supplementary Table 3**). PD-L1 was identified in 65.2% of CSCs versus in 28.6% of non-CSCs on average (*p <*0.05) and at a MFI of 8,716 versus 1,545 (**Figure 1**, **Supplementary Table 2**). An increase of >20% PD-L1 expression within the CSC group was appreciated in nearly every chordoma cell line evaluated (**Supplementary Table 3**). In CH22, the percent positivity of PD-L1 was comparable between groups (CSCs 16.2%, non-CSCs 20.1%). However, even in this cell line, the MFI of PD-L1 was notably greater in CSCs (3,777) as compared to non-CSCs (653) (**Supplementary Table 3**). All values included above are means of technical triplicates and representative of three independent experiments. Overall, the amount of CSCs analyzed *in vitro* within chordoma cell samples ranged from 352-11,100. The average number of CSCs analyzed per experiment using flow cytometry was 2533.

**Figure 2:**
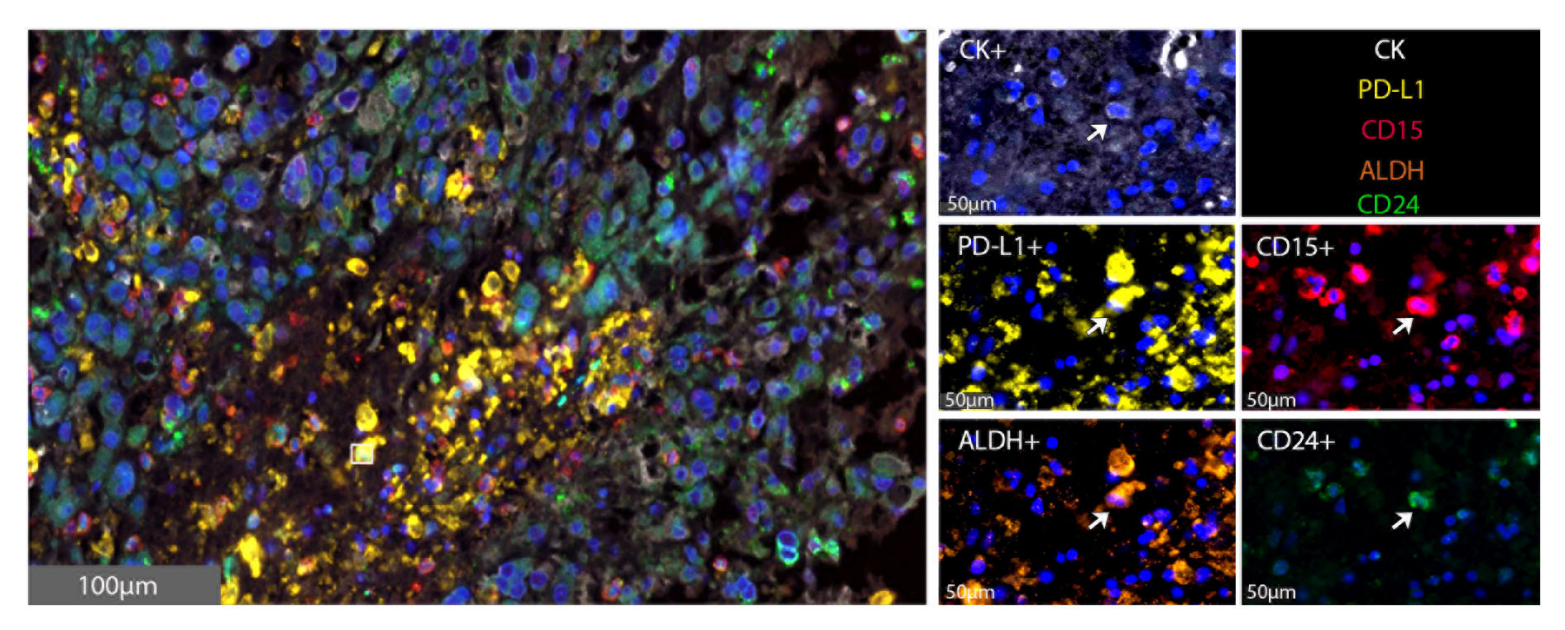
CSCs are identified within the chordoma tumor microenvironment. Representative photomicrographs from one chordoma of merged and single-color immunofluorescence images assessing the presence of CSCs with a validated panel of five biomarkers, PD-L1 (yellow, Opal 570), CD15 (red, Opal 690), ALDH (orange, Opal 620), CD24 (green, Opal 520), and CK (white, Opal 780). Multispectral immunofluorescence images are counterstained with DAPI. Co-localization of CD15, ALDH, and CD24 identified CSCs. Co-localization of these markers with PD-L1 identified PD-L1^+^ CSCs. CD15^+^CD24^+^ALDH^+^CK^+^ CSCs comprised 1.39% of all tumor cells. CK, cytokeratin; DAPI, 4′,6-diamidino-2- phenylindole; CSC, cancer stem cells.

*In vivo*, 18 resected chordoma samples were evaluated using multispectral immunofluorescence for CSCs, defined as cytokeratin positive chordoma cells that triple stained for CD15, CD24 and ALDH (**Figure 2**). Of all pooled chordoma cells across these tumor samples, CSCs comprised 1.39% of the population on average (**Table 2**). 75.18% of these CSCs were found to be PD-L1 positive (**Table 2**), a percentage notably higher than the 18.94% of general chordoma cells identified with PD-L1 positivity. No differences in chordoma cell PD-L1 positivity, CSC burden, or CSC PD-L1 positivity were identified with clinical correlations of patient age, tumor anatomic site of origin, disease stage, or radiotherapy treatment exposure (**Supplementary Figure 2**).

**Table 2:** CSC and PD-L1 percent positivity in chordoma cells.

	Chordoma Cells	PD-L1+ Chordoma Cells
Chordoma Cells		18.94%
CSCs (CD15^+^, CD24^+^, ALDH^+^)	1.39%	75.18%
NonCSCs	98.61%	15.18%

Chordoma samples were analyzed using the multispectral immunofluorescence technique. *In vivo* PD-L1 status is defined as percentage of CSCs identified within all 18 tumor microenvironments positive for PD-L1, using a signature of CD15^+^CD24^+^ALDH^+^PD-L1^+^CK^+^. CSCs, cancer stem cells.

**Figure 3:**
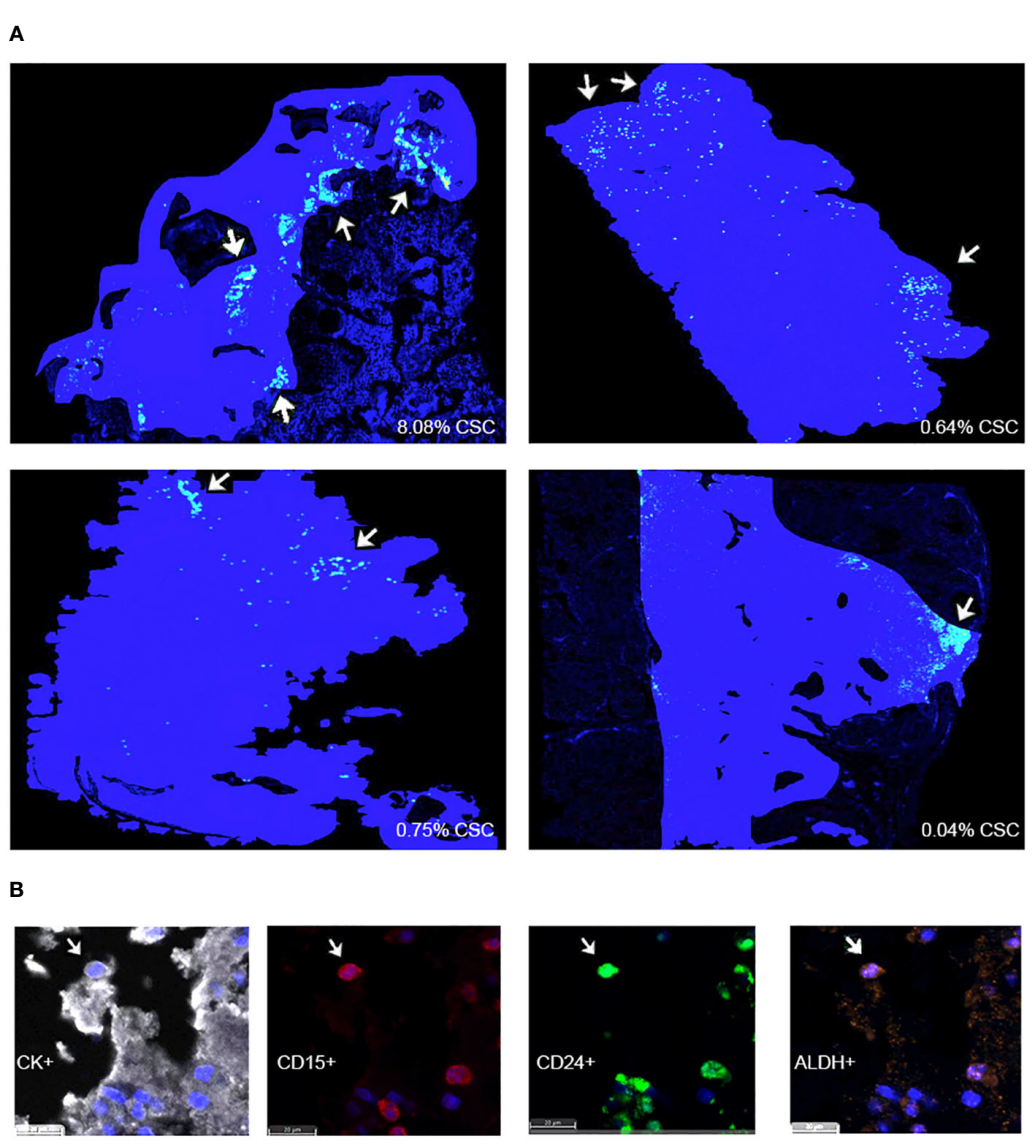
**(A)** HALO® density heat map analyses suggest that CSCs are found within clusters in four representative chordoma tumors. Solid blue patches delineate tumor areas included in the analysis. Grouped yellow and green dots, marked using white arrows, denote CSC clusters. **(B)** CK^+^ chordoma cells triple positive for cancer stem cell markers CD15, CD24, and ALDH are denoted CSCs. CSCs, cancer stem cells.

An infiltration spatial analysis of chordoma CSCs revealed an average distance of 71.51 μm (SD 73.40 μm) between CSCs and stroma. No difference in CSC distance to stroma was identified by patient age, tumor anatomic site of origin, disease stage, or radiotherapy treatment exposure (**Supplementary Figure 3**). A series of density heat maps created of each individual chordoma sample suggested a tendency of chordoma CSCs to cluster. This cellular subpopulation tended to be in groups, near in proximity to other chordoma CSCs. A series of four density heat maps with representative chordoma CSC clustering behavior is provided in **Figure 3**.

## Discussion

Chordoma is a devastating, locally invasive and destructive malignancy of the skull base, spine, and sacrum (2, 3, 5). Barriers to successful treatment lie in the tumor’s characteristic ability to resist chemoradiation and recur following surgical resection (2, 6). Chordoma cancer stem cells, a minority cellular subpopulation responsible for giving rise to and maintaining a proliferative tumor, are implicated in these aggressive attributes (12–14, 20, 21). This data demonstrates that NK activating ligand, tyrosine kinase, and PD-L1 markers, representative of potential immunotherapeutic targets, are favorably expressed by CSCs in six chordoma cell lines (**Figure 1**, **Table 2**). Furthermore, this study illustrates, for the first time, the *in vivo* presence and clustered spatial distribution of CSCs within the chordoma tumor microenvironment (**Figures 2**, **3**). No statistically significant differences in CSCs were appreciated by clinical correlates of patient age, tumor anatomic site of origin, disease stage, or radiotherapy in this patient cohort (**Supplementary Figure 2**). Chordoma CSCs quantified from cell culture using a CD24^+^CD133^+^ signature comprised <5% in all cell lines but one (**Table 1**), whereas CSCs appraised within imaged chordoma tumor samples using the CD15^+^CD24^+^ALDH^+^ signature comprised 1.39% of tumor cells (**Table 2**). Results confirm the sparse nature of this progenitor subpopulation and highlight the logistical challenge of its study in chordoma, a slow-growing tumor.

In this study, NK ligands B7H6, ULBP1, and MICA-B are expressed on the surface of a greater quantity of cells and at a significantly increased concentration within the CSC group as compared to the non-CSC group across six chordoma cell lines (**Figure 1**). Engaging NK cells against chordoma CSCs is important as NK cells possess a unique ability to exert cytolysis in the absence of antigen presentation, while also releasing cytokines and precipitating a coordinated organization of T cells (15, 34). B7H6 is part of the B7 family and binds natural cytotoxicity receptor NKp30 (27, 35). The NKp30-B7H6 complex precipitates a crucial NK cell-driven immune response against a tumor (27, 36). A phase I dose-escalation trial of BI 765049, a B7H6/CD3 T cell engager, with or without ezabenlimab (PD-1 inhibitor) for advanced solid tumors is ongoing (NCT04752215). UL16 binding protein 1 (ULBP1) and major histocompatibility complex (MHC) class I-related chain A and B polypeptides (MICA-B) are ligands of natural killer group 2 member D (NKG2D), an activating receptor of NK and cytotoxic T cells (29, 30, 37). MICA-B may prove useful as an immunotherapeutic target given its described localization to tumor cells while mostly sparing surrounding non-cancerous tissue (30). MICA-B is typically expressed on cell surfaces at low levels but is triggered by viral illness or malignant transformation (38). As the presence of MICA-B on malignant cells promotes an NK cell and cytotoxic T cell antitumor immune response, several strategies to augment this effect have been proposed, including employing MICA-B targeted antibodies to facilitate antibody dependent cellular cytotoxicity (ADCC) or to prevent the immune complex’s proteolytic shedding (39). One additional way to augment the NK and T cell coordinated response against CSCs, particularly in the setting of increased CSC NK ligand expression, could be to employ N803, an interleukin (IL)-15/IL-15-Rα superagonist (N803) previously shown by our group to enhance the killing of chordoma CSCs^15^. Of note, the authors only measured surface NK markers using both flow cytometry and mIF methods in the CSC and nonCSC groups rather than tumor-derived soluble NKG2D ligands, which are known to encourage immune suppression. Given the negative effect of soluble NKG2D ligands on anti-tumor NK cell function and on immune checkpoint blockade efficacy (40), the future study of these in chordoma may prove useful.

This data demonstrates that EGFR and HER2, both receptor tyrosine kinases (31), are expressed on the surface of nearly all chordoma CSCs and non-CSCs alike, but are identified at higher concentrations within the CSC group (**Figure 1**). Prior studies have described the amplification, transcriptional upregulation, and over-expression of EGFR in cancerous states and delineated this a biomarker of tumor resistance (41). In 2021, a review article described 14 globally approved EGFR reversible and irreversible inhibitors for anticancer treatment, including gefitinib and erlotinib, some of which may be considered in future studies of combinatorial immunotherapy against chordoma with a special focus on CSCs (42). In fact, a 2013 study has already demonstrated that erlotinib, a small molecule EGFR inhibitor, impeded the growth of a patient-derived chordoma in a xenograft model (43). Currently, three listed clinical trials are investigating EGFR inhibitors, including cetuximab (NCT05041127), afatinib (NCT03083678), and anlotinib hydrochloride versus imatinib (NCT04042597) for advanced, unresectable, or metastatic chordoma. Similarly, overexpression of human epidermal growth factor receptor 2 (HER2) in cancer has been linked to worse prognoses in breast, gastric, esophageal, ovarian, and endometrial malignancies (44). Monoclonal antibodies, trastuzumab and pertuzumab, and small molecule inhibitors, lapatinib, neratinib, and tucatinib developed to thwart HER2 (45) may be worth investigating in the context of chordoma CSCs.

PD-L1 was expressed by 65% of CSCs isolated from chordoma cell lines (**Figure 1**) and by 75% of CSCs recognized *in situ* within the chordoma tumor microenvironment (**Table 2**). Programmed cell death ligand 1 (PD-L1), expressed on many tumor cells, binds to programmed cell death 1 (PD-1) on T cells, B cells, NK cells, and myeloid derived suppressor cells (MDSCs) to drive an inhibitory response that suppresses T cell activity and fosters self-antigen tolerance. However, cancer cells exploit this pathway to circumvent the anti-tumor host immune response (33, 46). Preclinical evidence for the use of PD-L1 inhibitors suggests that chordoma cells express PD-L1, expression of which is further inducible by interferon gamma, and that chordoma CSCs are sensitive to PD-L1 inhibitor-mediated ADCC (15, 20). Current FDA approved PD-L1 inhibitors include avelumab, durvalumab, and atezolizumab for specific uses in advanced urothelial carcinoma, renal cell carcinoma, and non-small cell lung cancer (NSCLC), among others (46). Although pembrolizumab and nivolumab (PD-1 inhibitors) have been the more commonly studied immune checkpoint inhibitors in chordoma, durvalumab and FAZ053 (monoclonal antibodies with PD-L1 targets) were applied to limited chordoma treatment regimens with some clinical benefit (47, 48). To date, few ongoing clinical trials for PD-1/PD-L1 inhibitors include chordoma patients (NCT02936102, NCT02834013, NCT03190174). A neoadjuvant trial approach for PD-L1 blockade in chordoma would be interesting to determine whether targeting the chordoma PD-L1^+^ CSC subpopulation prior to standard of care surgical resection would help reduce disease recurrence.

Chordoma CSCs were found to take on a clustered pattern across *in vivo* tumor samples analyzed with multispectral immunofluorescence (**Figure 3**). Prior studies have similarly described a CSC niche, or specialized microenvironment within a tissue that is home to CSCs. It has been reported that small tumor cell clusters, surrounded by a favorable regional environment, may be present prior to the onset of metastasis (49). The average distance between chordoma CSCs and stroma across samples was found to be 71.51 μm. For reference, the cancer cell diameter has been reported at about 20 μm (50, 51), from which we can deduce that the mean CSC in our sample was seen at a distance equivalent to 3-4 cancer cell lengths from the tumor parenchyma periphery. This describes a target cell bundle within the chordoma tumor microenvironment to pursue using immunotherapeutic tools.

Therapeutics that minimize tumor bulk without effectively extinguishing progenitor CSCs, such as surgery or cytotoxic chemoradiation, encouragingly provide lesion regression but may fail to confer a long-term solution to halt chordoma regrowth and spread (12, 52). This underscores the importance of eliminating the CSC subpopulation for longitudinal treatment results. Ideally, a combination of therapy that quickly reduces tumor burden while ensuring enduring relapse prevention (12) would maximize patient survival. Reevaluating NK cell and T-cell immunotherapies against antigen targets on CSCs may be an avenue from which to explore this. Based on the study results presented here, the authors propose that the design of future immunotherapeutics for chordoma hone in on extinguishing clusters of CSCs, a strategy to be later combined with standard of care treatment. These efforts may begin with anti-PD-L1, anti-EGFR, or B7H6-enhancing techniques in the setting of data that supports CSC overexpression of these.

To our knowledge, this study offers the first detailed assessment of differential marker expression by CSC subpopulation status in chordoma. It also provides new insight into the *in situ* presence and arrangement of chordoma CSCs. The complementation of two methods, flow cytometry for which a precedent has been well-established, and mIF, a method much newer to chordoma, yields different lenses through which to view what have thus far been poorly understood chordoma CSCs. Despite the heterogeneous nature of chordoma and the differences in technique employed, the proportion of chordoma CSCs described in cell culture and tumor samples here were analogous, supporting our study results’ validity. The mIF technique used conferred strengths of single cell identification of rare cells within a tumor microenvironment, such as a CSC. Standardization of tumor staining using the PerkinElmer platform also minimized human error that accompanies staining samples by hand. Lastly, a 2012 paper by Yu et al. on CSCs proposed a need for future investigation into the link between treatment resistant CSCs and clinical outcomes, and it suggested starting with identification of CSC-specific surface markers (14). One particular strength of this study is the knowledge it supplies to begin answering this key question.

The use of dissimilar CSC definitions is a limitation of this study. Fujii et al. indicated increased CD15 and ALDH biomarkers in CD24^high^/CD133^high^ chordoma cells, termed the residential CSC population (20), substantiating the authors’ rationale for a CD24^+^CD133^+^ signature to discern CSCs *in vitro*. Furthermore, CD15 expression across all cell lines here was greater in CD24^high^/CD133^high^ chordoma cells than in the general chordoma cell population. However, significant staining challenges were encountered while validating the CD133 antibody for multispectral immunofluorescence (mIF), despite having tested multiple antibody versions from a variety of makers at a wide range of dilutions. Thus, the authors deemed a triplicate CD15^+^CD24^+^ALDH^+^ signature to be most specific and reliable in the quantification of CSCs within chordoma tumors. An inherent constraint of the mIF technique includes a maximum of six markers per antibody panel, four of which were reserved for CSC markers (CD15, CD24, ALDH) and cytokeratin, restraining the authors’ ability to efficiently corroborate flow cytometry findings of CSC surface biomarker expression in an *in vivo* setting. The use of emerging spatial transcriptomics, single cell sequencing, and other techniques providing increased simultaneous marker evaluation may aid in the further study of this tumor sub-population. While the mIF technique offers a useful *in situ* illustration of a tumor’s individual cells and compartments, it provides a still snapshot and cannot measure growth, division, or differentiation of CSCs over time. The authors were also unable to report average distances between CSCs due a spatial analysis software limitation. This study described no statistical differences in CSC surface marker expression or spatial distribution by tumor characteristic or treatment regimen. A small sample size (n= 18) of tumors with retrospectively-collected clinical data, comprised only of two recurrent tumors and with no metastatic tumors, restricted the authors’ ability to draw meaningful conclusions regarding potentially informative correlations between CSC properties and patient status. The future study of CSCs in recurrent and metastatic chordomas, for which a more robust set of clinical data is required, represents a principle area of interest given the discussed associations between tumor-sustaining cells and recurrence and survival outcomes. Finally, a prior study of this same patient cohort characterized the myeloid cell, T cell, and natural killer cell compartments of the chordoma tumor immune microenvironment (TIME) using mIF (26). This study’s tumor images and those of the TIME analysis were unable to be superimposed to explore relationships between immune cells implicated in an anti-tumor response and the chordoma CSC subpopulation. However, this represents a future direction for this work.

## Conclusion

This study provides a deeper understanding of the chordoma cancer stem cell surface phenotype and *in situ* organization. The sparing of CSCs with currently available antitumor therapies may account for continued high recurrence and metastasis rates. Thus, developing therapeutic regimens against tumor-initiating and sustaining CSCs may improve longitudinal chordoma control. Potential immunotherapeutic targets, such as PD-L1, NK binding ligands, and tyrosine kinases, are elucidated here for the elimination of chordoma CSC clusters.

## Data availability statement

The raw data supporting the conclusions of this article will be made available by the authors, without undue reservation.

## Ethics statement

The studies involving humans were approved by the National Institutes of Health Institutional Review and the Johns Hopkins Institutional Review Board. The studies were conducted in accordance with the local legislation and institutional requirements. Written informed consent for participation was not required from the participants or the participants’ legal guardians/next of kin because the National Institutes of Health Institutional Review Board provided an exemption and all Johns Hopkins participants had written consent for the use of tissue for research.

## Author contributions

DL: Validation, Writing – review & editing, Writing – original draft, Visualization, Project administration, Methodology, Investigation, Formal analysis, Data curation, Conceptualization. KF: Writing – review & editing, Methodology, Investigation, Formal analysis, Data curation, Conceptualization. MP: Writing – review & editing, Supervision, Resources. YR: Formal analysis, Writing – review & editing, Investigation, Data curation. JK: Writing – review & editing, Conceptualization. WL: Writing – review & editing, Investigation, Data curation. DP: Conceptualization, Writing – review & editing. CA: Writing – review & editing, Visualization, Resources, Data curation, Conceptualization. GG: Resources, Writing – review & editing, Conceptualization. JG: Writing – review & editing, Conceptualization. JH: Writing – review & editing, Visualization, Supervision, Resources, Data curation, Conceptualization. NL: Writing – review & editing, Visualization, Supervision, Resources, Funding acquisition, Data curation, Conceptualization.
