# Supplementary material for: Chordoma cancer stem cell subpopulation characterization may guide targeted immunotherapy approaches to reduce disease recurrence
Source: Front Oncol. 2024 Apr 29;14:1376622. doi: 10.3389/fonc.2024.1376622 (PMC11089222; doi:10.3389/fonc.2024.1376622)
Supplement: Supplementary file 1 [file DataSheet_1.docx]

**Supplementary Figure 1.** Chordoma cell flow cytometry gating strategy


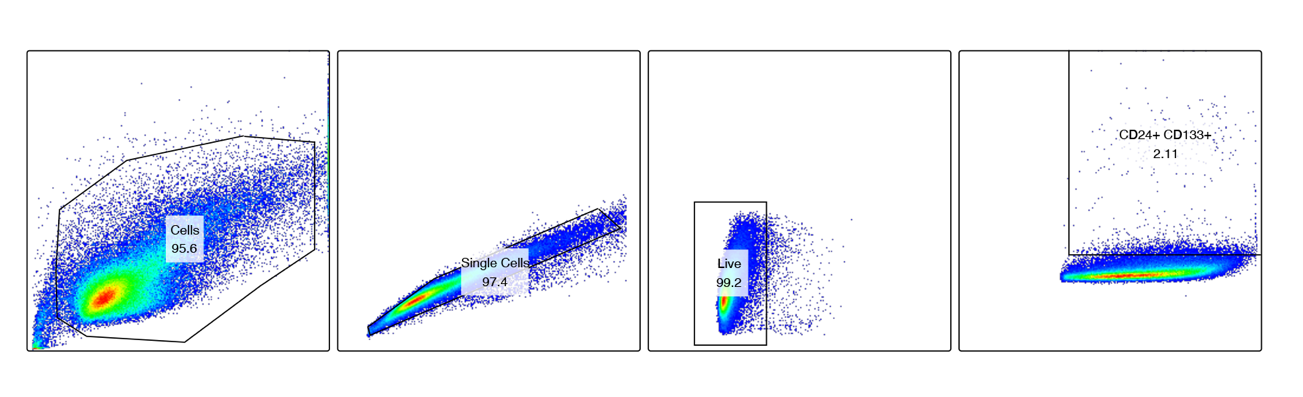


Supplementary Figure 1. A) Flow cytometry gating strategy demonstrating isolation of CD24^+^CD133^+^ CSCs in chordoma cell line MugChor1. CSCs, cancer stem cells.

**Supplementary Figure 2.** Proportions of PD-L1^+^ chordoma cells, total CSCs, and PD-L1^+^ CSCs by Clinical Correlate


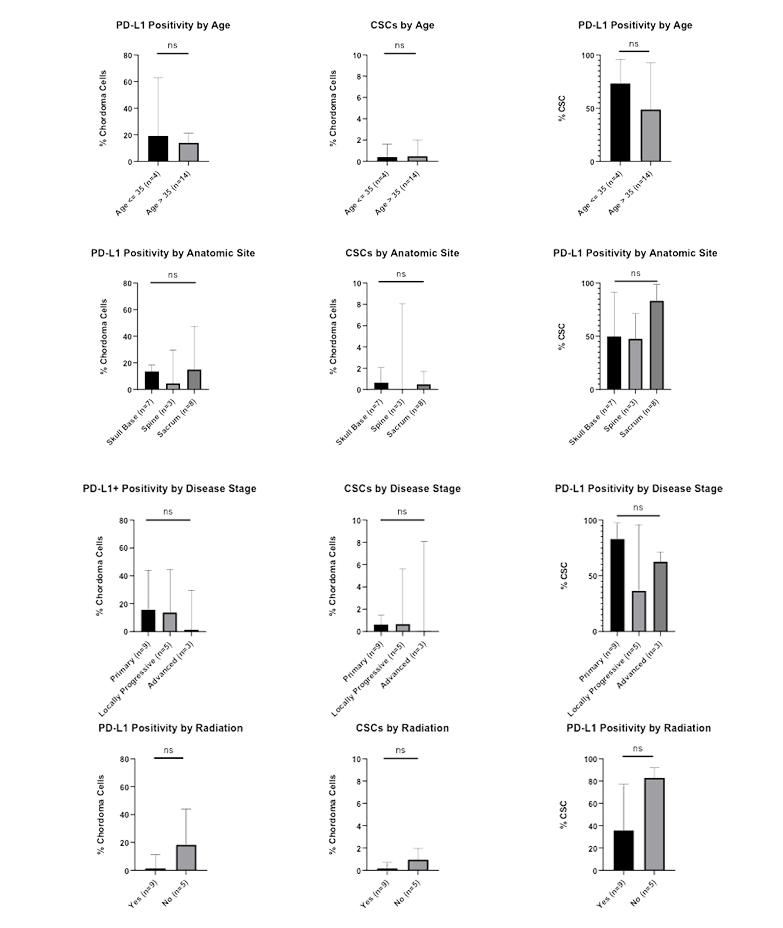


Supplementary Figure 2. Proportions of PD-L1^+^ chordoma cells, total CSCs, and PD-L1^+^ CSCs do not significantly differ by age, anatomic site of origin, disease stage, or radiation therapy. A comparison by age was made between chordoma patients ≤35 years old and >35 years old (n=18). A comparison by radiation was made between chordoma patients who received a degree of pre- or postoperative radiation therapy (n=9) versus not (n=5). Both analyses were completed with a Mann Whitney test. A comparison by anatomic site of origin was made among chordomas originating from skull base (n=7), spine (n=3), or sacrum/coccyx (n=8). A comparison by disease stage was made among chordomas resected at primary (n=9), locally aggressive (n=5), and advanced (n=3) stage. Both analyses were completed with a Kruskal-Wallis analysis. A p value threshold <0.05 defined statistical significance. CSC, cancer stem cell; ns, non-significant.

**Supplementary Figure 3.** CSC Distance to Stroma by Clinical Correlate

Supplementary Figure 3. Infiltration analyses show that the average CSC distance to stroma (µm) does not significantly differ by age, anatomic site of origin, disease stage, or radiation therapy. A comparison by age was made between chordoma patients ≤35 years old and >35 years old (n=18). A comparison by radiation was made between chordoma patients who received a degree of pre- or postoperative radiation therapy (n=9) versus not (n=5). Both analyses were completed with a Mann Whitney test. A comparison by anatomic site of origin was made among chordomas originating from skull base (n=7), spine (n=3), or sacrum/coccyx (n=8). A comparison by disease stage was made among chordomas resected at primary (n=9), locally aggressive (n=5), and advanced (n=3) stage. Both analyses were completed with a Kruskal-Wallis analysis. A p value threshold <0.05 defined statistical significance. CSC, cancer stem cell; ns, non significant.

**Supplementary Table 1.** CSC Multispectral Immunofluorescence Antibody Panel


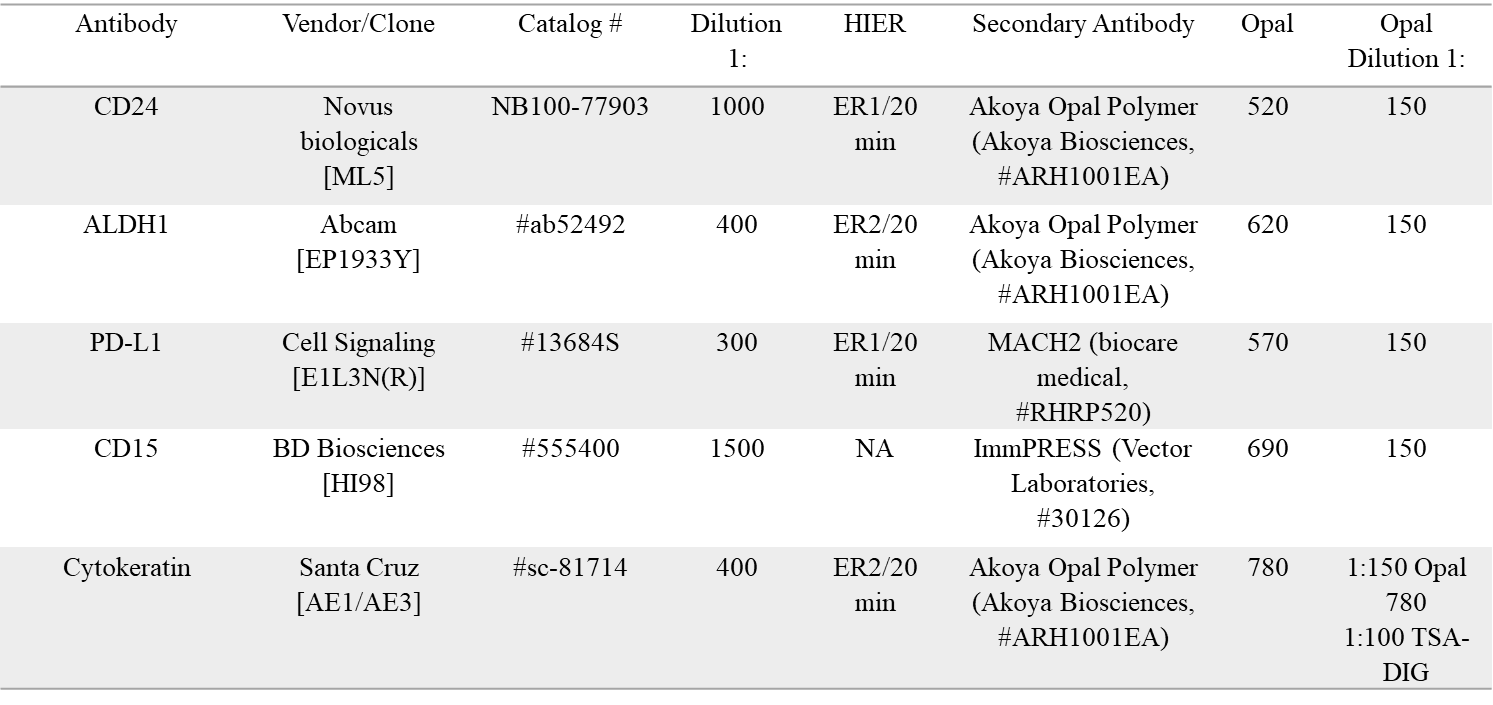


Supplementary Table 1. Multispectral immunofluorescence antibody panel used to assess chordoma CSCs i*n vivo*.

**Supplementary Table 2.** Chordoma CSC Characterization by Surface Marker

**
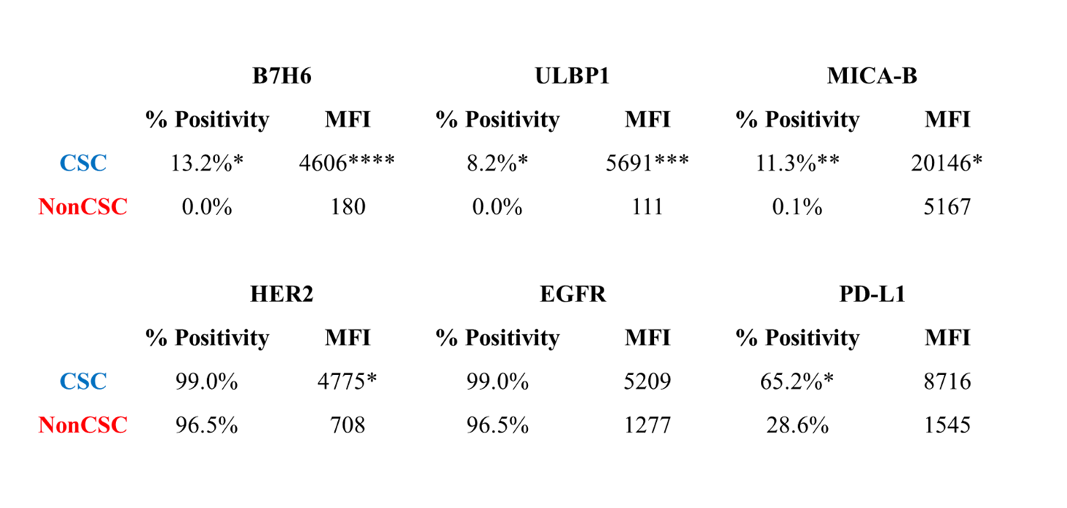
**

Supplementary Table 2. *In vitro* chordoma CSC characterization by surface markers of B7H6, ULBP1, MICA-B, HER2, EGFR, and PD-L1, measured both by percent positivity (top) and MFI (bottom). This is the same data presented in Figure 1 shown here in table form. Average values across six cell lines, collected via flow cytometry, are shown. Values for CSCs (blue) and non-CSCs (red) were compared using student’s t tests with a significance threshold of p <0.05. **p*≤0.05, ***p*≤0.01, ****p*≤0.001, *****p*≤0.0001. CSC, cancer stem cells; MFI, mean fluorescence intensity.

**Supplementary Table 3.** Chordoma CSC Characterization by Surface Marker in All Cell Lines

Supplementary Table 3. *In vitro* chordoma CSC characterization by surface markers of B7H6, HER2, MICA-B, ULBP1, EGFR, and PD-L1, measured both by percent positivity (left) and MFI (right). Values across six cell lines collected via flow cytometry are shown. Any increase in expression of >20% positivity or a 5-fold MFI increase in CSCs (blue) compared to NonCSCs (red) are shown bolded and in orange. Each value depicted here is the mean of technical triplicates within one experiment, and results are representative of three independent experiments. CSC, cancer stem cells; MFI, mean fluorescence intensity.
